# Nicotinamide adenine dinucleotide phosphate oxidase 4 in lung disease: a review of its biology and therapeutic potential

**DOI:** 10.3389/ebm.2026.10941

**Published:** 2026-06-12

**Authors:** Yilin Wang, Tianru Ben, Jianjiang Fang, Zengpan Li, Jinhua Ding, Liyan Xu, Kai Lin, Li Jiang

**Affiliations:** 1 The Fourth Clinical College of China Medical University, Shenyang, China; 2 The First Clinical College of China Medical University, Shenyang, China; 3 Department of Emergency, The Affiliated Lihuili Hospital of Ningbo University, Ningbo, China; 4 Department of Breast Surgery, The Affiliated Lihuili Hospital of Ningbo University, Ningbo, China; 5 Department of General Practice, The Affiliated Lihuili Hospital of Ningbo University, Ningbo, China; 6 Department of Geriatrics, The Affiliated Lihuili Hospital of Ningbo University, Ningbo, China

**Keywords:** acute lung injury, chronic obstructive pulmonary disease, idiopathic pulmonary fibrosis, nicotinamide adenine dinucleotide phosphate oxidase, pulmonary hypertension

## Abstract

Nicotinamide adenine dinucleotide phosphate oxidase 4 (NOX4) is a constitutively active enzyme that primarily produces hydrogen peroxide, a reactive oxygen species (ROS) with diverse cellular functions. While initially recognized for its role in oxidative stress, emerging evidence suggests that NOX4 plays a pivotal role in the pathogenesis of various lung diseases. This review delineates the structure characteristics of NOX4, emphasizing how its domain organization underlies a distinctive mode of molecular regulation. It further discusses current knowledge on the biological functions of NOX4-derived oxygen species, including their roles in modulating inflammation, cell death pathways, oxygen sensing, nuclear signaling, and metabolic reprogramming. Through these interconnected processes, NOX4 is positioned as a central mediator linking redox imbalance to cellular dysfunction. In addition, the contribution of NOX4 to the pathogenesis of major lung diseases, including idiopathic pulmonary fibrosis (IPF), chronic obstructive pulmonary disease (COPD), asthma, acute lung injury/acute respiratory distress syndrome (ALI/ARDS), and pulmonary hypertension are critically evaluated. Emerging therapeutic strategies targeting NOX4 are also discussed, together with key challenges associated with clinical translation, including isoform specificity, off-target effects, and tissue-selective delivery. Overall, this review provides an integrated framework for understanding NOX4 biology across multiple levels and highlights its potential as a therapeutic target in lung disease.

## Impact statement

Lung diseases driven by oxidative stress remain a major cause of morbidity and mortality, yet therapies targeting reactive oxygen species have largely failed due to a lack of specificity. This review is important because it focuses on NOX4, a distinct and constitutively active source of oxidants that plays a central role in lung injury, inflammation, fibrosis, and vascular remodeling. By integrating structural biology, regulatory mechanisms, and disease-specific functions, this work advances the field by explaining why NOX4 acts not merely as a damage-inducing enzyme but as a context-dependent signaling hub. The review brings together emerging evidence on nuclear NOX4, metabolic reprogramming, and regulated protein turnover—areas that have not previously been synthesized in a lung-focused framework. This integrated perspective reframes NOX4 as a tractable, disease-modifying therapeutic target and provides a clear rationale for selective NOX4 inhibition in chronic and acute lung diseases.

## Introduction

Reactive oxygen species (ROS) are a double-edged sword in biological systems. At low concentrations, they act as crucial signaling molecules involved in various physiological processes, including cell proliferation [1–3], differentiation [4, 5], and immune responses [6–8]. However, excessive ROS production, termed oxidative stress, contributes to cellular damage and is implicated in the pathogenesis of numerous diseases [9]. NADPH oxidases (NOXs) are a family of enzymes specifically dedicated to generating ROS, with seven isoforms (NOX1-5 and DUOX1-2) identified in mammals. Among these, NOX4 stands out due to its unique characteristic of being constitutively active and primarily producing hydrogen peroxide (H_2_O_2_) rather than superoxide [10]. The lung, being constantly exposed to environmental oxidants and inflammatory stimuli, is particularly vulnerable to oxidative stress [11]. Consequently, NOX enzymes, including NOX4, are highly expressed in various lung cell types, such as epithelial cells [12, 13], fibroblasts [14, 15], endothelial cells [16–18], and smooth muscle cells [19, 20]. The intricate and often conflicting roles of NOX4 in lung health and disease have garnered significant research interest, paving the way for potential therapeutic interventions. This review aims to consolidate the current knowledge regarding NOX4’s multifaceted involvement in lung diseases, providing a comprehensive overview of its pathogenic mechanisms and therapeutic implications. To better understand how NOX4 exerts these diverse biological effects, it is essential to first examine its unique structural features, which distinguish it from other NOX isoforms and underline its constitutive activity.

## Molecular structure of NOX family and specific features of NOX4

**FIGURE 1:**
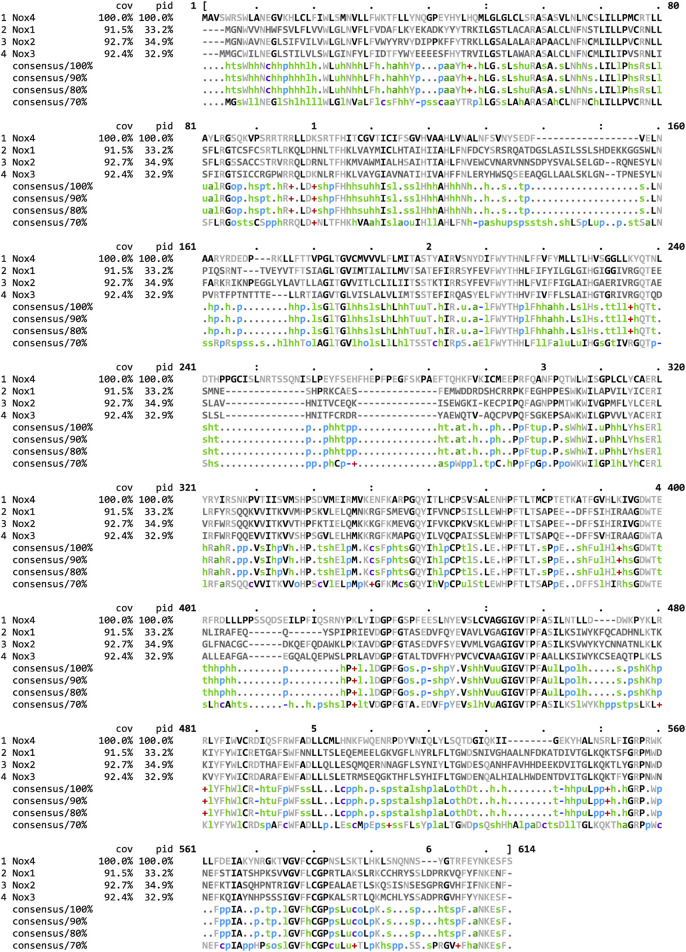
NOX family proteins alignment. The alignment of NOX1–NOX4 proteins reveals a high degree of conservation across transmembrane regions and key functional motifs, particularly within the NADPH oxidase core domains. NOX4, serving as the reference sequence, shows 100% identity and coverage, while Nox1, Nox2, and Nox3 exhibit ∼91%–93% identity but only ∼33%–35% pairwise identity, indicating substantial divergence in non-conserved regions. The transmembrane helices and FAD/NADPH-binding motifs are notably preserved, suggesting functional constraints across the family. However, the N-terminal and C-terminal regions display marked variability, likely reflecting isoform-specific regulatory roles or subcellular targeting. Consensus sequences at varying identity thresholds (70%–100%) highlight conserved residues critical for enzymatic activity and membrane integration, while gaps and substitutions pinpoint regions of evolutionary flexibility. This alignment underscores both the structural integrity and functional diversification within the NOX family.

The NADPH oxidase (NOX) family are membrane-bound enzymes that share a conserved core architecture but differ significantly in their regulatory domains and activation mechanisms. As illustrated in Table 1, all NOX isoforms possess transmembrane domains with six α-helices and two heme-binding sites, and a cytosolic dehydrogenase domain that binds flavin adenine dinucleotide (FAD) and nicotinamide adenine dinucleotide phosphate (NADPH) for electron transfer [21, 22]. However, their regulatory domains vary: NOX1, NOX2, and NOX3 require cytosolic subunits like p47phox and p67phox for activation [23]; NOX5 contains EF-hand calcium-binding domains, making it calcium-sensitive [24, 25]. In contrast, NOX4 lacks these regulatory domains, making it constitutively active—it does not require cytosolic activators or calcium for ROS production [26, 27]. These structural differences underpin the functional diversity of NOX isoforms in physiological and pathological contexts. In the following sections, we will focus primarily on NOX4 and compare its structure features with those of NOX1-3, which are more loosely related to it than NOX5 or DUOX1/2. As shown in Figure 1, NOX1–3 share ∼91%–93% identity with NOX4 overall, their pairwise identity is much lower (∼33%–35%), reflecting notable divergence in non-conserved regions. This divergence is most pronounced in the N- and C-terminal regions, which may contribute to isoform-specific regulatory mechanisms or differences in subcellular localization. By contrast, the preservation of transmembrane helices and FAD/NADPH-binding sites indicates that the structural framework required for catalytic activity is maintained across isoforms. These observations suggest that the NOX family combines a conserved enzymatic core with variable terminal regions that confer functional diversification. NOX4 contains highly conserved sequences and domains that are essential for its unique structure, constitutive activity, and function as a generator of hydrogen peroxide. These conserved regions, shaped by evolution, distinguish NOX4 from other members of the NOX family and are crucial for its biological roles.

**TABLE 1:** NOX4 domains and residue localization.

Region	Residue range	Description
N-terminal tail	1–65	Cytosolic; may influence localization and transcriptional regulation
Transmembrane Helix I	66–85	First membrane anchor; part of the conserved catalytic core
Extracellular Loop 1	86–105	Short loop; may modulate ROS accessibility or isoform-specific interactions
Transmembrane Helix II	106–125	Anchors the protein; contributes to electron flow
Intracellular Loop 1	126–145	Connects helices; may interact with cytosolic partners or stabilize structure
Transmembrane Helix III	146–165	Conserved helix; part of the redox center
Extracellular Loop 2	166–185	Larger loop; potential role in ROS diffusion or extracellular signaling
Transmembrane Helix IV	186–205	Anchors the protein; contributes to FAD positioning
Intracellular Loop 2	206–225	May stabilize FAD/NADPH binding domains
Transmembrane Helix V	226–245	Final membrane anchor; links to extracellular loop 3
Extracellular Loop 3	246–265	Often glycosylated; may regulate extracellular ROS release.
Transmembrane Helix VI	266–285	Final helix; connects to cytosolic catalytic domains
FAD-binding domain	286–400	Essential for electron transfer; highly conserved across NOX isoforms
NADPH-binding domain	401–560	Drives ROS production; includes Rossmann fold-like motifs
C-terminal tail	561–578	Cytosolic; may regulate activity or interact with scaffolding proteins

### Transmembrane domains

The transmembrane domains are conservative among NOX family. All NOX family members possess six transmembrane helices that span the cellular membrane. Similarly, NOX4 transmembrane domains span from amino acid 100 to 300. These helices are crucial for anchoring the enzyme within internal membranes, such as the endoplasmic reticulum (ER) and nuclear membrane. Within the helices, two heme groups, which are essential for electron transfer, are buried in helices 3 and 5. These hemes serve as a critical bridge in the electron transport chain from the FAD cofactor [28, 29].

### Loops connecting the transmembrane helices

Between the six transmembrane helices, five extracellular and intercellular loops are formed, labeled A through E. These loops, along with the N- and C-termini, are crucial for the enzyme’s function, stability, and unique product specificity. The orientation of the loops alternates between the intracellular (cytosolic) and extracellular (luminal) sides of the membrane. Loops A, C, and E are extracellular, while loops B and D are intracellular. Loop A, connecting transmembrane helices 1 and 2, is on the extracellular or luminal side of the membrane. Its role is not as extensively studied as the other loops, but it is believed to be important for the overall structural integrity and correct folding of the NOX4 enzyme. As an extracellular loop, it’s involved in shaping the environment on the side where oxygen is reduced. Loop B is located between transmembrane helices 2 and 3. In human NOX4, this region contains a conserved polybasic sequence. The B-loop is a critically important as its function is to serve as a key interface between the transmembrane domain and the dehydrogenase (DH) domain of the protein [30, 31]. While other NOX isoforms use this loop to interact with cytosolic regulatory subunits like p47phox, NOX4 does not. Instead, its B-loop binds directly to the C-terminal DH domain, which contains the binding sites for FAD and NADPH [3, 31–33]. This internal binding is a key reason for NOX4’s constitutive activity, as it provides a stable connection that keeps the enzyme in an “on” conformation without needing external signals [10]. Loop C extracellularly connects transmembrane helices 3 and 4. Like loop A, it is involved in maintaining the overall protein structure and ensuring the correct orientation of the transmembrane helices [34]. It plays a role in the formation of the channel through which electrons are transferred, and oxygen is reduced, but its specific function is less defined compared to the E-loop. Loop D is located between transmembrane helices 4 and 5 and is on the cytosolic side of the membrane. It’s crucial for the interaction with p22phox [35]. Mutational analysis of NOX4 has revealed that the D-loop is critical for the proper heterodimerization of the NOX4-p22phox complex, which is necessary for NOX4 stability and activity [36]. Mutations in this loop can abolish the functional interaction with p22phox [36]. Loop E is the final extracellular loop, connecting transmembrane helices 5 and 6. In NOX4, it is significantly longer than the E-loop in other NOX isoforms. It is the key determinant of why NOX4 produces H_2_O_2_ as its primary product [37], unlike other NOX enzymes that mainly produce superoxide. A highly conserved histidine residue in this loop is thought to be strategically positioned to act as a proton donor. This unique structural feature allows the E-loop to promote the rapid dismutation of the newly formed superoxide radical to hydrogen peroxide before the ROS product leaves the enzyme, effectively “trapping” and converting it. This makes the E-loop central to NOX4’s characteristic signaling properties.

### DH domain

Located at the C-terminus and facing the cytosol, the DH domain is the catalytic core of NOX4. This domain contains binding sites for both FAD and NADPH [31–33]. The DH domain is intrinsically activated in NOX4, meaning it doesn’t require additional cytosolic regulatory subunits (unlike other NOX isoforms such as NOX1-3 that need proteins like p47 and p67 for activation). This inherent activity allows NOX4 to constitutively transfer electrons from NADPH to FAD.

Also part of the DH domain, a region from 401 to 560 amino acids is responsible for binding NADPH [31, 33], the electron donor for the enzyme. Electrons from NADPH are transferred to the FAD, initiating the electron transport chain that ultimately leads to oxygen reduction and H_2_O_2_ production. Once bound, NADPH transfers two electrons to the FAD cofactor, which is bound to a β-barrel fold within FAD-binding subdomain ranging from amino acids of 286–400. The electrons are then passed from FAD to a heme group in the transmembrane domain of the enzyme [31]. This transfer is facilitated by the interaction between the DH domain and the B-loop, a polybasic region connecting transmembrane helices. This interaction helps bring the FAD and heme groups into close proximity, enabling electron transfer [30]. While the structural organization of NOX4 explains its intrinsic enzymatic activity, its biological impact is primarily determined by tight regulation of its expression and stability under physiological and pathological conditions.

## Molecular regulation of NOX4

The molecular regulation of NOX4 is a complex process that primarily occurs at the level of its expression and protein stability, rather than through a rapid, on-demand activation like other NADPH oxidases. This constitutive activity makes its regulation distinct and crucial for controlling its cellular effects.

### Regulation by transforming growth factor-β (TGF-β)

TGF-β signaling is a potent inducer of NOX4 expression [38–42]. This is a key mechanism in the pathogenesis of fibrotic diseases, as increased NOX4 expression drives the pro-fibrotic signaling cascade. When the cytokine TGF-β1 binds to its receptors, it triggers a phosphorylation cascade that activates Smad2 and Smad3 [38, 43, 44]. These proteins then form a complex with the common mediator Smad4 and translocate into the nucleus. In the nucleus, the Smad complex binds to specific DNA sequences called Smad Binding Elements (SBEs), located within the promoter of the NOX4 gene [45–48]. This binding initiates the recruitment of co-activators and the transcriptional machinery, leading to a robust increase in NOX4 gene transcription. This creates a powerful positive feedback loop, as NOX4-derived ROS, in turn, can activate latent TGF-β1, further amplifying the fibrotic response.

### Regulation by hypoxia

Under hypoxia, the transcriptional regulation of NOX4 is primarily controlled by Hypoxia-Inducible Factor 1 (HIF-1) [49, 50]. During hypoxia, the HIF-1α subunit is stabilized and translocated to the nucleus, where it heterodimerizes with HIF-1β [51]. This complex then binds to specific hypoxia-responsive elements (HREs) in the promoter regions of target genes [52, 53]. The NOX4 promoter contains HREs, allowing HIF-1α to directly bind and drive its transcription [20, 49]. This HIF-1-mediated upregulation of NOX4 in hypoxia can contribute to various pathologies, including pulmonary hypertension (PH) [20]. The expression of NOX4 is also regulated by common transcription factors like AP-1 and Sp1, which can respond to a variety of upstream signals, including growth factors and stress [54]. AP-1, a dimer composed of Fos and Jun proteins, and Sp1 are known to bind to consensus sequences within the NOX4 promoter [46]. Their binding can either synergize with or act independently of other transcription factors to modulate NOX4 expression. The coordinated action of these and other transcription factors allows for a precise and nuanced control of NOX4 levels in response to diverse cellular signals.

### Epigenetic regulation

NOX4 expression can also be regulated by epigenetic mechanism. This regulation primarily occurs through DNA methylation, histone modifications, and non-coding RNAs, which collectively modulate the accessibility of the NOX4 gene to the transcriptional machinery. Hypermethylation of the NOX4 promoter is generally associated with gene silencing or a decrease in NOX4 expression [55, 56]. The addition of methyl groups physically obstructs the binding of transcription factors, preventing the initiation of transcription. Conversely, promoter hypomethylation can lead to the “un-silencing” of the gene, resulting in its upregulation. The acetylation of histones, mediated by histone acetyltransferases (HATs), generally loosens chromatin structure, making the promoter region of the NOX4 gene more accessible and thereby promoting its transcription [57]. Interestingly, histone deacetylases (HDACs) remove these acetyl groups, leading to a condensed chromatin state, also increase NOX4 expression [58–60]. This discrepancy adds complexity to the regulation of NOX4. The use of HDAC inhibitors, for example, has been shown to reduce NOX4 expression in some contexts [59, 61]. Similar to DNA methylation, the methylation of histones can have both activating and repressive effects on gene expression, depending on the specific histone residue and the number of methyl groups. For example, trimethylation of histone H3 at lysine 4 (H3K4me3) is often associated with active transcription [62–65], while trimethylation at lysine 27 (H3K27me3) is linked to gene silencing [66]. These marks likely influence NOX4 expression, though the precise patterns are still under investigation. MicroRNAs are small, non-coding RNAs that regulate gene expression post-transcriptionally by binding to specific messenger RNA (mRNA) molecules, leading to their degradation or translational repression. This mechanism is also a key player in the epigenetic control of NOX4. A number of miRNAs have been identified that directly target the NOX4 mRNA. For instance, miR-9-5p has been shown to suppress NOX4 expression by binding to its 3′-untranslated region, leading to mRNA degradation [67]. The dysregulation of this and other NOX4-targeting miRNAs can contribute to a variety of pathological conditions [68–70], including fibrosis [67, 71], by altering the delicate balance of NOX4 levels. The ability of miRNAs to fine-tune NOX4 expression provides a sophisticated layer of negative regulation that complements its other control mechanisms.

### Regulation by ubiquitination

NOX4 protein levels can also be regulated by ubiquitination and subsequent proteasomal degradation. The ubiquitination of NOX4 is a multi-step enzymatic cascade catalyzed by a trio of enzymes. E1 (ubiquitin-activating enzyme), E2 (ubiquitin-conjugating enzyme), and E3 (ubiquitin ligase). The E3 ubiquitin ligase is the most important component, as it provides the specificity by recognizing the target protein and catalyzing the transfer of ubiquitin. For NOX4, specific E3 ligases recognize key motifs on the protein, leading to the formation of polyubiquitin chains, which serve as a signal for degradation by the 26S proteasome. The specificity of NOX4 ubiquitination is largely determined by a few well-characterized E3 ubiquitin ligases and their associated adaptor proteins. Hic-5 is a major negative regulator of NOX4 that acts as an adaptor protein. In response to oxidative stress (often generated by NOX4 itself), Hic-5 undergoes a conformational change that allows it to bind directly to NOX4. Hic-5 then recruits a specific E3 ubiquitin ligase, leading to the polyubiquitination and subsequent degradation of NOX4 [72]. This forms an elegant and rapid negative feedback loop, preventing excessive ROS production and helping to restore redox balance. CHIP is a well-known E3 ubiquitin ligase involved in protein quality control. It partners with molecular chaperones like Hsp70 to recognize misfolded or damaged proteins. Studies have shown that CHIP can bind to and ubiquitinate NOX4, targeting it for proteasomal degradation [73]. This function ensures that improperly folded or aggregated NOX4 is removed from the cell, maintaining a healthy pool of functional enzyme.

## The expanding biological roles of NOX4

The diverse and often contradictory functions of NOX4—from promoting cell survival to inducing cell death—are a testament to the context-dependent nature of its signaling. This intricate regulation is primarily mediated by the ability of H_2_O_2_ to reversibly oxidize specific cysteine residues on target proteins, thereby modulating their activity.

### The role in inflammation and cell death pathways

**FIGURE 2:**
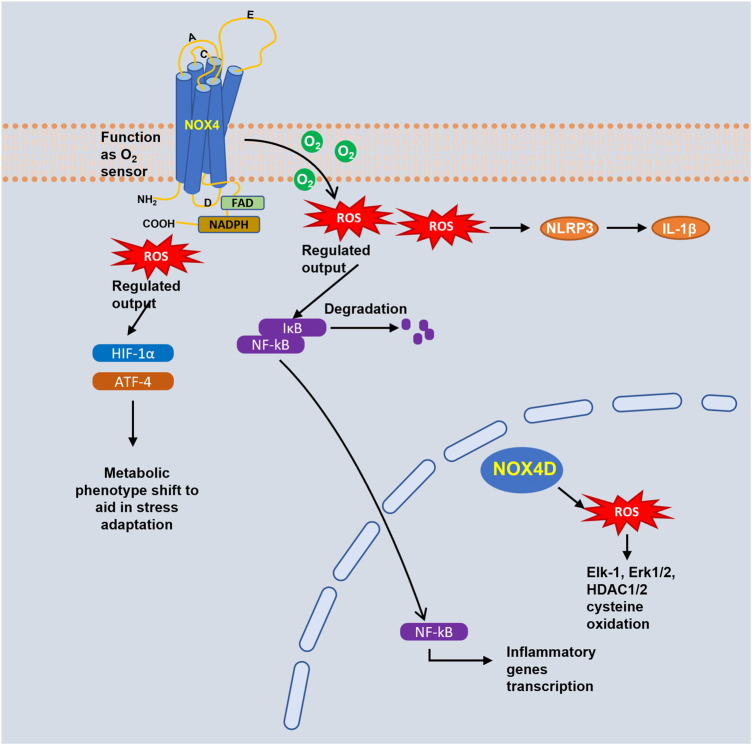
NOX4 signaling and its role in cellular stress and inflammation. The diagram illustrates the function of NADPH oxidase 4 (NOX4) as an oxygen sensor, producing reactive oxygen species (ROS) upon activation. NOX4, through the NADPH binding site and FAD domain, generates ROS that regulate various cellular outputs. These include activation of transcription factors such as HIF-1α and ATF-4, which drive a metabolic phenotype shift to aid in stress adaptation. ROS also lead to the degradation of IκB, resulting in NF-κB activation and the transcription of inflammatory genes. Additionally, ROS regulate the NLRP3 inflammasome and the release of interleukin-1β (IL-1β), further promoting inflammatory responses. ROS-induced modifications of proteins, such as cysteine oxidation of Elk-1, Erk1/2, and HDAC1/2, also contribute to inflammation-related gene transcription.

NOX4 is a key orchestrator of the inflammatory response, particularly through its influence on the NFκB pathway, as seen in LPS-mediated activation [74]. As shown in Figure 2, in its inactive state, the NFκB transcription factor is sequestered in the cytoplasm by its inhibitor, IκB. NOX4-derived ROS facilitates the phosphorylation and subsequent degradation of IκB, thereby promoting NFκB to translocate to the nucleus [75, 76]. In the nucleus, NFκB drives the transcription of a vast array of pro-inflammatory genes, including those encoding cytokines like IL-8 and TNF-α. This molecular link positions NOX4 as an important amplifier of both acute and chronic inflammatory conditions. Furthermore, NOX4 has been shown to regulate pyroptosis [77–79], a highly inflammatory form of programmed cell death, by promoting a redox environment for NLRP3 inflammasome activation [78, 80, 81]. Under certain circumstances NOX4-derived ROS can contribute to NLRP3 inflammasome formation, leading to the cleavage and release of pro-inflammatory cytokines like IL-1β and IL-18. This novel function positions NOX4 as a central mediator of inflammation and cell death in the context of various inflammatory diseases. Recent studies have established a direct link between NOX4 and non-apoptotic forms of cell death, specifically ferroptosis [82–84]. Ferroptosis is a form of iron-dependent cell death characterized by the accumulation of lipid peroxides. While NOX4 is not considered a primary trigger of ferroptotic cell death, its constitutive production of H_2_O_2_ contributes to ferroptosis by depleting the antioxidant glutathione (GSH) [82, 83], thereby tipping the balance toward oxidative damage and lipid peroxidation. In this context, NOX4 acts to sensitize cells to ferroptotic stimuli and amplify oxidative damage rather than directly executing the ferroptotic program. Beyond its role in inflammatory signaling and cell death, NOX4 also functions as a sensor of environmental cues, particularly oxygen availability.

### NOX4 as an oxygen sensor

In addition to its roles in inflammation and cell death, NOX4 functions as a molecular oxygen sensor, linking oxygen tension to cellular signaling [10, 85]. NOX4 functions as a biochemical oxygen rheostat, translating oxygen levels into redox signals via H_2_O_2_ production. This makes it a critical player in both normal physiological and pathophysiological conditions like fibrosis, NOX4 has a much higher Michealis constant for oxygen compared to NOX2 (18% vs. 2%–3%) [10], meaning it operates efficiently across a wide physiological range of oxygen tensions. This property allows it to act as a graded sensor, adjusting hydrogen peroxide output in proportion to oxygen availability. This oxygen-sensing capability enables NOX4 to regulate processes like hypoxia response [86], angiogenesis [87–89], cellular differentiation [76, 90–94], especially in tissues like the kidney, vasculature, and heart. NOX4-derived ROS influences the activity of key ion channels, such as the KCNK3/TASK-1 potassium channel [95]. In vascular smooth muscle cells, NOX4’s regulation of these channels is critical for controlling pulmonary vascular tone and can contribute to conditions like PH [95]. Furthermore, NOX4-derived ROS can influence the stability and transcriptional activity of HIF-1α, the master regulator of the hypoxic response, thereby regulating a cell’s metabolic and genetic adaptation to low oxygen [96, 97]. In addition to cytoplasmic and mitochondrial signaling, emerging evidence highlights a distinct role for NOX4 within the nucleus.

### The nuclear function of NOX4

While NOX4 is widely recognized for its roles in the ER and mitochondria, a unique and critical function of the enzyme is its presence and activity within the nucleus and nuclear membrane. A pivotal discovery was the identification of a 28-kDa splice variant of NOX4, termed NOX4D, which lacks transmembrane domains and localizes to the nucleus and nucleolus [98, 99]. This variant was found in vascular smooth muscle cells [98] and other cell types [99, 100], where it actively generates ROS and influences nuclear signaling pathways. Here, NOX4 operates as a direct regulator of the nuclear microenvironment by H_2_O_2_ in close proximity to the cell’s genetic material. This targeted ROS production serves as a potent signaling mechanism that directly influences both transcriptional and epigenetic processes. On a molecular level, the H_2_O_2_ can reversibly oxidize key redox-sensitive cysteine residues on nuclear proteins, including transcription factors such as Elk-1 and ERK1/2, thereby modulating their ability to bind to DNA and regulate gene expression. This allows NOX4 to act as a direct molecular switch, controlling genes related to cell proliferation, differentiation, and stress responses as shown in Figure 2.

Furthermore, nuclear NOX4’s activity can influence chromatin remodeling by affecting the function of enzymes like histone deacetylases (HDACs), providing an additional layer of epigenetic regulation that can alter DNA accessibility and long-term gene expression patterns. This mechanism is particularly relevant in conditions like cellular senescence [101, 102] and chronic inflammation. For instance, in lung fibroblasts, high levels of NOX4 are associated with an increase in the active histone mark H4K16Ac [103] and a decrease in the repressive mark H4K20Me3 [57], which supports an active transcriptional state of the NOX4 gene itself [57, 103]. NOX4 can also influence histone methylation. For example, in the context of inflammation, NOX4-derived nuclear ROS can oxidize and inhibit the activity of HDAC1/2, a class of enzymes that can interact with and regulate histone marks [104]. This “chromatin remodeling” can lead to altered gene expression of pro-inflammatory cytokines, driving the inflammatory response.

This localized production of ROS in the nucleus also carries a dual risk: while it is vital for signaling, an excessive or dysregulated amount can lead to oxidative DNA damage and genomic instability. Overexpression of NOX4D induces γ-H2A phosphorylation [105, 106], a marker of DNA-double strand breaks, linking NOX4 to genomic instability. This effect is dependent on the integrity of the NDAPH-binding domain, as a single amino acid substitution abolishes ROS production and downstream signaling. These spatially distinct functions of NOX4 are further complemented by its ability to regulate cellular metabolism.

### Metabolic reprogramming

NOX4 has been newly identified as a regulator of cellular metabolism, going beyond its previously known effects on mitochondria. It is now understood to modulate pathways such as fatty acid oxidation and glucose metabolism [107–112]. By altering the redox state of key metabolic enzymes, NOX4 influences a cell’s metabolic preferences, contributing to metabolic reprogramming. This role is particularly significant in cancer [113] and diabetes [110, 114], where metabolic shifts are central to disease progression. NOX4-derived H_2_O_2_ can activate or inhibit redox-sensitive transcription factors. For instance, in some cancers, NOX4 deficiency leads to the upregulation of MYC and Nrf2 [113, 115, 116], which are master regulators of cell proliferation and metabolic homeostasis. This promotes a more aggressive, glycolytic phenotype. Conversely, in other contexts, NOX4-generated ROS activate pro-survival pathways like HIF-1 alpha [97, 117] and ATF4 (activating transcription factor 4) [118–120], which aid in stress adaptation (Figure 2). Meanwhile, NOX4 can directly alter how cells use different fuel sources. In the heart, NOX4-mediated signaling shifts metabolism away from glucose oxidation and towards fatty acid oxidation [108]. This is mediated through the hexosamine biosynthetic pathway (HBP), which enhances protein O-GlcNAcylation that helps the heart adapt to stress and maintain its energetic status.

Collectively, these diverse biological functions position NOX4 as a central regulator of redox-dependent signaling. Dysfunction of these processes can lead to persistent inflammation, aberrant cell death, and pathological tissue remodeling. In the lung, such alterations are closely linked to the development and progression of multiple respiratory diseases.

## The pathophysiological role of NOX4 in lung diseases

**FIGURE 3:**
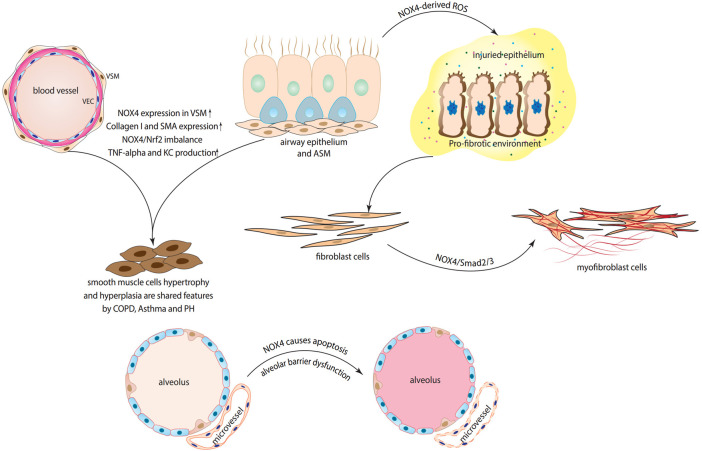
NOX4 contributes to the pathogenesis of multiple lung disorder by targeting a broad spectrum of structural and resident cells within the lung. NOX4 ability to drive oxidative stress, cellular injury and inflammatory signaling is central to disease initiation and progression. Key cellular targets of NOX4 include airway epithelial cells, airway and vascular smooth muscle cells, fibroblasts, and endothelial cells, where it modulates redox-sensitive pathways and influence cell survival, differentiation, and function. In chronic lung disease such as IPF, COPD, asthma, and PH, NOX4-dependent mechanisms show substantial overlap. These include enhanced proliferation, hyperplasia, and hypertrophy of airway and vascular smooth muscle cells, contributing to airway narrowing and increased vascular resistance. In parallel, NOX4 promotes fibroblast activation, differentiation into myofibroblasts, and excessive extracellular matrix deposition, which are hallmark features of fibrotic remodeling in IPF. Notably, similar fibroblast activation pathways are also implicated in airway remodeling in asthma, highlighting shared pathogenic mechanisms across these conditions. In contrast, ARDS is mechanically distinct from these chronic disorders. ARDS is characterized by acute and widespread injury to the alveolar epithelium and pulmonary microvascular endothelium. In this setting, NOX4 directly contributes to epithelial and endothelial cell damage, leading to increased permeability and breakdown of the alveolar barrier. This disruption results in protein-rich fluid leakage into the alveolar space.

Chronic respiratory diseases represent a significant global health burden, driving intensive research efforts to uncover the precise cellular and molecular mechanisms behind their progression. ROS play a dual role in lung biology, acting as crucial signaling molecules in healthy tissue while contributing to cellular damage and remodeling during disease pathogenesis. NOX4 has emerged as a central, yet complex, player in the progression of chronic respiratory illnesses. Understanding the dysregulated expression and function of NOX4 is critical, as growing evidence links its activity to the underlying pathology of devastating conditions such as Idiopathic Pulmonary Fibrosis (IPF) and Chronic Obstructive Pulmonary Disease (COPD) (see Table 2). Through modulation of redox-sensitive signaling pathways, NOX4 influences diverse aspects of lung cells, including airway epithelial cells, smooth muscle cells, fibroblasts, and endothelial cells (See Figure 3). These cells represent substantial mechanistic overlap of lung disease. The following sections examine the role of NOX4 in the pathophysiology of chronic and acute lung diseases.

**TABLE 2:** The role of NOX4 in pulmonary disease.

Disease	NOX4-involved mechanisms of pulmonary disease
IPF	1. NOX4-mediated fibroblast differentiation into myofibroblasts via ROS-dependent activation of Smad2/3 signaling [15, 103, 128–130] 2. NOX4-induced TGF-β1 expression [131, 132] 3. NOX4-mediated early epithelial cell death in response to injury [12, 133] 4. NOX4 promoting myofibroblast differentiation, deposition of ECM [134]
COPD	1. NOX4 expression is upregulated in ASM and PASMCs by TGF-β, TNF-α [134, 138, 139] 2. NOX4-derived ROS promote collagen I deposition and α-SMA expression [134, 139] 3. NOX4 drives vascular wall thickening and smooth muscle proliferation in distal pulmonary arteries [138] 4. NOX4 activates NF-κB, promoting pro-inflammatory cytokines expression [141, 142] 5. NOX4/Nrf2 imbalance exacerbate mitochondrial damage and mitophagy [143]
Asthma	1. Airway smooth muscle cells proliferation, hypertrophy [149], and hypersensitivity [19] 2. Elevated NOX4 in cilial epithelial cells reduces cilial beat frequency [150] 3. NOX4 upregulation is involved in mitochondrial damage–induced apoptosis in bronchial epithelial cells [69] 4. In fibroblasts, PI3K-dependent TRPV4 activation mediates NOX4 upregulation, which is required for TGF- β1–induced lung fibroblast differentiation through MRTF-A and PAI-1 [151]
ALI	1. Upregulated NOX4 in alveolar macrophages, endothelia cells and epithelial cells [13, 17, 155] 2. NOX4-derived H_2_O_2_ activates NF-κB pathway [74, 156] 3. NOX4 contributes directly to alveolar epithelial can endothelial cells apoptosis [12, 13, 17, 104, 157] 4. NOX4 impairs phagocytic function of macrophages, preventing effective cleaning apoptotic cells and repairing [76, 158]
PAH	1. Marked overexpression of NOX4 in remodeled pulmonary arteries [20, 166] 2. Increased NOX4 activates PDGFR-β/Akt pathway leading to activated growth and survival pathways in PASMCs [167, 168] 3. NOX4 compromises endothelial cell integrity, promotes apoptosis by reducing NO bioavailability [169, 170] 4. NOX4-derived H_2_O_2_ impairs angiogenesis and promotes vascular remodeling [172]

### NOX4 in IPF

IPF is a chronic, progressive, and often fatal lung disease characterized by the excessive accumulation of extracellular matrix (ECM) components, leading to irreversible scarring and loss of lung function [121]. While the exact etiology of IPF remains elusive, mounting evidence points to a critical role for oxidative stress in its pathogenesis [122, 123]. The lung’s continuous exposure to environmental insults and its high oxygen tension makes it particularly vulnerable to oxidative stress [124]. The initial injury to the delicate alveolar epithelium is a hallmark event, which triggers a cascade of events. The damaged epithelial cells release pro-fibrotic mediators, attracting and activating fibroblasts [125–127]. This leads to the overproduction and deposition of ECM components, such as collagen and fibronectin. Among the molecular drivers of IPF, NOX4 has emerged as a central regulator of fibrogenic signaling. NOX4-derived ROS play a pivotal role at every stage of this cascade, acting not as mere byproducts of cellular activity but as crucial signaling molecules that drive the disease forward. The first major study implicating NOX4 in IPF demonstrated that NOX4 expression is significantly upregulated in pulmonary fibroblasts from IPF patients, especially in response to TGF-β1 [15]. This study showed that NOX4 mediates fibroblast differentiation into myofibroblasts via ROS-dependent activation of Smad2/3 signaling. These findings established NOX4 as a critical mediator of fibrogenic transformation. Further validation came from studies showing that NOX4 is also upregulated in fibroblastic foci, the pathological hallmark of IPF(128), and that its inhibition can reverse fibrotic phenotypes *in vitro* and *in vivo* [103, 128–130]. There are several mechanisms underlying NOX4 contribution to IPF. NOX4 enhances TGF-β1-induced expression of profibrotic genes, including α-smooth muscle actin (α-SMA) and collagen [131, 132]. NOX4 has been implicated in epithelial cell death, particularly in response to injury [12, 133], which is considered an early and crucial step in the development of lung fibrosis [125, 126]. This epithelial damage can then trigger fibroblast activation and subsequent ECM deposition. By promoting myofibroblast differentiation, NOX4 directly contributes to the overproduction and deposition of ECM components like collagen and fibronectin, driving the fibrotic process [134].

Therapeutic potentials: Recent efforts have led to the development of highly selective NOX4 inhibitors, which demonstrate efficacy in reversing fibrosis in aged mouse models [135]. These compounds inhibit ROS production, myofibroblast differentiation, and collagen synthesis. Metformin, an AMPK activator, has been shown to suppress NOX4 expression and attenuate lung fibrosis in bleomycin-induced models [128]. This highlights the potential of metabolic modulators in targeting NOX4-mediated pathways. Studies have explored the role of NOX4 in aging-related fibrosis, showing that its sustained upregulation in aged lungs contributes to persistent fibrotic remodeling [136]. Epigenetic regulation of NOX4 may offer new therapeutic angles. Emerging strategies aim to combine NOX4 inhibitors with Nrf2 activators to simultaneously suppress oxidant production and boost antioxidant defenses [135]. Preclinical models have demonstrated proof-of-concept for NOX4-targeted therapies, and clinical trials are anticipated to evaluate safety and efficacy in IPF patients.

While NOX4-driven fibrotic remodeling has been most extensively characterized in IPF, several of these pathogenic mechanisms are also shared with COPD. Both diseases are marked by persistent oxidative stress, chronic inflammation, and aberrant tissue remodeling. These overlapping pathological features suggest that NOX4 may function as a common upstream regulator linking redox imbalance to both fibrotic and inflammatory process across distinct pulmonary disease contexts. In this regard, emerging evidence supports a similar role for NOX4 in the pathogenesis of COPD.

### NOX4 in COPD

COPD, primarily caused by cigarette smoke exposure, is characterized by chronic inflammation, airflow limitation, and progressive lung damage [137]. Oxidative stress is a major contributor to COPD pathophysiology. Among the enzymatic sources of ROS, NOX4 has gained attention due to its constitutive activity and ability to generate H_2_O_2_, contributing to redox imbalance and tissue injury [138]. Multiple studies have demonstrated that NOX4 expression is significantly upregulated in the lungs of COPD patients, particularly in airway smooth muscle (ASM) and pulmonary artery smooth muscle cells (PASMCs) [134, 138, 139]. This upregulation correlates with disease severity, airway remodeling, and PH, a common complication of advanced COPD [138]. *In vivo* and *in vitro* studies confirm that NOX4 expression is inducible by pro-inflammatory cytokines such as TGF-β1 and TNF-α, both of which are elevated in COPD [134, 140]. NOX4-derived ROS promote collagen I deposition and α-SMA expression in bronchial smooth muscle cells, contributing to ASM hypertrophy and hyperplasia [134, 139]. This remodeling is mediated through noncanonical TGF-β signaling, involving p38 MAPK and Akt pathways, which are activated in response to cigarette smoke and inflammatory stimuli [134]. In COPD patients with PH, NOX4 expression is elevated in distal pulmonary arteries, where it drives vascular wall thickening and smooth muscle proliferation [138]. This remodeling is associated with reduced pulmonary function and right ventricular hypertrophy, as shown by cardiac MRI studies [138]. NOX4 contributes to oxidative stress by generating ROS that damage lipids, proteins, and DNA. In COPD lungs, this is evidenced by increased malondialdehyde and decreased superoxide dismutase levels [138]. NOX4 also modulates the NF-κB pathway, promoting the expression of pro-inflammatory cytokines such as TNF-α and KC [141, 142]. Recent studies show that NOX4/Nrf2 imbalance exacerbates mitochondrial damage and mitophagy, particularly in response to environmental pollutants like PM2.5, increasing susceptibility to acute exacerbations of COPD (AECOPD) [143].

Therapeutical potentials: The traditional use of broad-spectrum antioxidants in COPD has yielded limited success [144], highlighting the need for more specific therapies. Targeting NOX4 offers a rational approach because it strikes at a key upstream driver of multiple pathologies. Instead of simply scavenging ROS, a NOX4 inhibitor can prevent its production at the source, thereby simultaneously blocking inflammation, cell death, and remodeling. The most advanced and well-studied specific inhibitor is setanaxib (GKT137831) [145]. This small-molecule compound acts as a dual inhibitor of both NOX4 and NOX1. Its mechanism involves blocking the transfer of electrons at the catalytic site of the enzyme, thereby suppressing the generation of ROS. Setanaxib has been investigated in clinical trials for other fibrotic diseases like primary biliary cholangitis and liver stiffness (ClinicalTrials.gov ID: NCT05014672, phase II), diabetic kidney disease (ClinicalTrias.gov ID: NCT02010242, phase II) and IPF (ClinicalTrias.gov ID: NCT03865927, phase II), and these trials have provided valuable data on its safety and pharmacokinetics. However, large-scale, dedicated clinical trials to definitively prove its efficacy in a broad COPD population have yet to be completed. The potential for NOX4 inhibition to be a disease-modifying therapy for COPD, rather than just a symptomatic treatment, remains a subject of intense research.

### NOX4 in asthma

Asthma and chronic obstructive pulmonary disease (COPD) have traditionally been viewed as distinct airway disorders; however, accumulating evidence indicates that they share several common pathological features, particularly in chronic and severe disease states [146, 147]. Both conditions are characterized by persistent airway inflammation, oxidative stress, and structural remodeling, which collectively contribute to airflow limitation [147]. A central shared mechanism is oxidative stress, driven by both environmental exposures (e.g., allergens, pollutants, cigarette smoke) and endogenous sources such as NADPH oxidases.

Asthma has long been associated with elevated oxidative stress [148]; however, the involvement of NOX4 in asthma was not recognized until 2007, when it was first shown to mediate TGF-β1–induced airway smooth muscle cells proliferation and hypertrophy [149]. Subsequent genome-wide microarray analyses revealed increased NOX4 expression in airway smooth muscle cells from patients with asthma, which was further validated by PCR and protein assays [19]. Functionally, airway smooth muscle cells isolated from asthmatic individuals exhibits enhanced agonist-induced contraction, a response that is attenuated by NOX4 knockdown as well as by pharmacological inhibitors such as diphenyleneiodonium and apocynin, indicating a critical role for NOX4 in airway smooth muscle hyperresponsiveness.

Later studies confirmed that elevated NOX4 expression in asthma is not restricted to airway smooth muscle cells but is also present in other airway cell types. A hallmark of asthma, particularly neutrophilic asthma, is impaired mucociliary clearance. In this context, NOX4 protein levels are significantly higher in airway epithelial cells from patients with neutrophilic asthma compared with those from non-neutrophilic asthma patients and healthy controls. Importantly, these patients exhibit reduced ciliary beat frequency, a key indicator of ciliary function, which can be restored by setanaxib [150].

More recent clinical observations demonstrate a significant correlation between TLR4 and NOX4 mRNA expression in patients with inflammatory asthma, further supporting a role for NOX4 in disease-associated inflammation. In addition, mechanistic studies using IL-13–treated BEAS-2B cells show that NOX4 upregulation is associated with mitochondrial damage–induced apoptosis and decreased levels of miR-182-5p, a microRNA that negatively regulates NOX4 expression by targeting the 3′ untranslated region of its mRNA [69].

Beyond epithelial and smooth muscle dysfunction, NOX4 also plays a central role in airway remodeling through regulation of fibroblast activation. Asthma-associated remodeling is characterized by persistent myofibroblast differentiation driven by TGF-β1, mechanical stress, and oxidative signaling. Recent study has identified the mechanosensitive ion channel TRPV4 as a critical upstream regulator that integrates these signals via a TRPV4–NOX4 axis [151]. Activation of TRPV4 promotes NOX4 expression and ROS generation, which are required for TGF-β1–induced lung fibroblast differentiation through myocardin-related transcription factor-A (MRTF-A) and plasminogen activator inhibitor-1 (PAI-1). Mechanistically, both TRPV4 and NOX4 are activated downstream of PI3K signaling, and coordinated input from TRPV4 and Rac is necessary for NOX4 upregulation. Importantly, inhibition or genetic deletion of TRPV4 suppresses NOX4 expression and ROS production, thereby attenuating fibroblast activation and allergen-induced airway remodeling *in vivo*. Consistent with these findings, fibroblasts derived from asthmatic patients exhibit higher NOX4 expression compared to those from non-asthmatic controls.

### NOX4 in acute lung injury (ALI)

ALI and ARDS are life-threatening conditions characterized by severe inflammation, increased vascular permeability, and impaired gas exchange [152]. Oxidative stress is a key mediator of lung damage in these conditions [153, 154]. In the context of ALI/ARDS, NOX4 is significantly upregulated in key cell types within the lung, including alveolar macrophages [155], endothelial cells [17] and epithelial cells [13]. Its activity contributes to the disease through several interconnected molecular mechanisms. NOX4-derived H_2_O_2_ acts as a potent pro-inflammatory signal. It triggers the activation of crucial signaling pathways, most notably the NF-κB pathway [74, 156]. By promoting the degradation of the inhibitory IκB protein, NOX4 allows NF-κB to translocate to the nucleus and drive the transcription of numerous pro-inflammatory genes [74]. This results in the massive production of inflammatory cytokines like IL-8 and TNF-alpha which recruit and activate more immune cells, creating a destructive feedback loop that sustains the inflammatory response. A hallmark of ALI/ARDS is the extensive damage and death of the alveolar-capillary barrier’s cellular components. NOX4 contributes directly to the apoptosis of both alveolar epithelial and endothelial cells [12, 13, 17, 157]. This widespread cell death compromises the integrity of the lung barrier, leading to the leakage of protein-rich fluid into the alveoli, a phenomenon known as alveolar flooding. This fluid accumulation is a primary cause of the severe hypoxemia and respiratory failure seen in patients. In addition to causing injury, NOX4 can also hinder the body’s natural processes of resolution and repair. It can impair the phagocytic function of macrophages [76, 158], preventing them from effectively clearing apoptotic cells and cellular debris from the airspaces. This failure to clear dead cells sustains inflammation and prevents the lung from properly repairing its damaged tissue, contributing to the poor prognosis of the disease.

### NOX4 in PH

PH is a severe, debilitating disease defined by a sustained elevation in pulmonary arterial pressure, leading inevitably to right ventricular failure [159]. The underlying structural pathology involves progressive, irreversible pulmonary arterial smooth muscle cell (PASMC) proliferation [160], endothelial dysfunction [161, 162], and excessive vascular remodeling [163–165]. This destructive cascade is highly dependent on chronic, dysregulated oxidative stress, making NOX4 a critical enzymatic hub driving the disease. The hallmark of PH is the muscularization and thickening of the pulmonary arterial wall. NOX4 is markedly overexpressed in the remodeled pulmonary arteries of both human PH patients and in various preclinical animal models [20, 166]. The H_2_O_2_ generated by NOX4 serves as a potent signaling molecule that initiates growth and survival pathways in PASMCs. Specifically, in models of PH induced by chronic intermittent hypoxia (CIH)—relevant to conditions like obstructive sleep apnea—increased NOX4 expression has been directly correlated with the pathological activation of the platelet-derived growth factor receptor beta (PDGFR-beta) and its downstream signaling cascade, including the PI3K/Akt kinase [167, 168]. This sustained activation accelerates PASMC proliferation and migration, driving the shift from a contractile to a synthetic phenotype and ultimately increasing vascular resistance.

The vascular endothelium is highly vulnerable to NOX4-induced stress. Pathological increases in NOX4 activity compromise endothelial cell integrity, promoting apoptosis and leading to a loss of barrier function and impaired vasodilation, primarily due to reduced nitric oxide (NO) bioavailability [169, 170]. Furthermore, NOX4 dysregulation is not limited to adult PH. While some neonatal models of PH induced by chronic hypoxia in piglets show unchanged NOX4 expression [171], other models, such as those for persistent pulmonary hypertension of the newborn (PPHN) in lambs, clearly demonstrate increased NOX4 and its critical regulatory subunit p22 expression [172]. This elevation is linked to NOX4-derived H_2_O_2_ mediated signaling that impairs angiogenesis (new blood vessel formation) and promotes vascular remodeling via NF-κB activation and subsequent cyclin D1 upregulation, suggesting its involvement in congenital and developmental forms of the disease.

The consistent identification of NOX4 as a pathological effector positions it as a highly attractive therapeutic target for PH. Specific small-molecule inhibitors of NOX4, such as setanaxib, have been rigorously tested in preclinical PH models [173]. These studies consistently demonstrate that NOX4 inhibition can significantly attenuate PASMC proliferation, reduce overall vascular remodeling, and decrease measured pulmonary arterial pressure. Crucially, this pathogenic role is further underscored by findings that established pharmacological agents known to reverse PH, such as Rosiglitazone, achieve their therapeutic effects, in part, by suppressing both NOX4 expression and the resulting superoxide production in the lung vasculature [174]. Thus, targeting NOX4 offers a promising, disease-modifying strategy to counteract the fundamental drivers of oxidative stress and remodeling in PH.

## Discussion

The conceptual transition of NOX4 from a general marker of oxidative stress to a discrete, targetable mediator of lung pathology represents an important shift in our understanding of redox biology in pulmonary disease. As highlighted in this review, NOX4 is not merely a passive source of reactive oxygen species, but rather a context-dependent regulator that integrates environmental and cytokine-driven signals into sustained cellular responses. In particular, its role in promoting myofibroblast activation, (epithelial–mesenchymal transition) EMT, and persistent tissue remodeling underscores its contribution to both inflammatory injury and fibrosis. Among the pulmonary diseases discussed, IPF and COPD appear to be the most promising candidates for NOX4-targeted interventions, given the consistent upregulation of NOX4 observed in human tissues and their strong association with fibrotic and airway remodeling processes. In contrast, the role of NOX4 in diseases with more heterogeneous inflammatory profiles, such as asthma and ARDS, remains less clearly defined.

Considering the failure of non-specific antioxidant approaches, there is the need for a more targeted and mechanistically informed strategy for NOX4 modulation. A central challenge lies in defining the therapeutic “redox window,” whereby pathological H_2_O_2_ production is selectively suppressed without disrupting physiological signaling required for normal cellular function. To address these challenges, several priorities should be emphasized. First, setanaxib is NOX1 and NOX4 inhibitor, it is needed to improve specificity and minimizing interference with NOX2-dependent host defense mechanisms. Second, integrative therapeutic approaches, including the combination of NOX4 inhibitors with established anti-fibrotic agents (e.g., nintedanib or pirfenidone) or modulators of antioxidant pathways such as Nrf2 activators, may provide synergistic benefits by simultaneously limiting oxidant production and enhancing endogenous defense systems. Third, the cell type-specific roles of NOX4 in different lung compartments need to be completely defined and the relationship between NOX4 expression or activity and disease endotypes, progression, and therapeutic response needs to be established. Finally, the long-term consequences of sustained NOX4 inhibition, especially with respect to physiological redox signaling, host defense, and tissue homeostasis, require careful evaluation in human studies. Addressing these gaps will be essential for translating mechanistic insights into safe and effective disease-modifying therapies.
